# Relationship of OqxAB efflux pump to antibiotic resistance, mainly fluoroquinolones in *Klebsiella pneumoniae*, isolated from hospitalized patients

**DOI:** 10.22038/IJBMS.2022.67095.14714

**Published:** 2023-01

**Authors:** Fereshteh Amereh, Mohammad Reza Arabestani, Leili Shokoohizadeh

**Affiliations:** 1 Department of Microbiology, School of Medicine, Hamadan University of Medical Sciences, Hamadan, Iran; 2 Infectious Disease Research Center, Hamadan University of Medical Sciences, Hamadan, Iran

**Keywords:** Antimicrobial, Ciprofloxacin, Efflux, *K. pneumoniae*, Pump, Resistance

## Abstract

**Objective(s):**

This research was designed to study the prevalence of OqxAB efflux pump genes and also to investigate the relationship between efflux pump and resistance to antibiotics, especially to fluoroquinolones, evaluate the expression levels of OqxAB genes, and molecular typing of *Klebsiella pneumoniae* isolated from hospitalized patients in Hamadan hospitals, west of Iran.

**Materials and Methods:**

In a cross-sectional study, 100 clinical strains of *K. pneumoniae* were isolated from hospitalized patients in three major teaching hospitals from January to June 2021. The antibiotic susceptibility of isolates was evaluated by the disk-diffusion agar method. The frequency of genes encoding *oqx*A and *oqx*B of efflux pump genes was investigated by PCR, and the expression of the *oqx*A efflux pump gene was investigated by the Real-time PCR method. The genetic relationship of *K. pneumoniae* isolates was analyzed by the Enterobacterial Repetitive Intergenic Consensus (ERIC)-PCR technique.

**Results:**

According to our results, the multi-drug resistance phenotype (MDR) in 65% and high prevalence resistance to ciprofloxacin in 89% of *K. pneumoniae* isolates was detected. The higher prevalence of *oqx*A (95%) and *oqx*B (98%) was also detected. There was a significant relationship between ciprofloxacin resistance and the *oqx*B gene as well as between ceftriaxone and chloramphenicol resistance and the *oqx*A gene. The expression of the *oqx*A gene was higher in ciprofloxacin-resistant isolates.

**Conclusion:**

The results of this study suggest a potential reservoir for the spread of OqxAB genes among hospital-acquired bacteria. Infection control strategies should be used prudently to reduce the spread of resistant strains of *K. pneumoniae* in hospitals.

## Introduction

*Klebsiella pneumoniae* is one of the most important nosocomial pathogens that cause various infections in the urinary tract, bloodstream, and respiratory tract. This bacterium has the ability to develop resistance to cephalosporins, carbapenems, fluoroquinolones, aminoglycosides, and even polymyxins (1, 2). *K. pneumoniae* may also produce a thick layer of extracellular biofilm, which helps them adhere to various surfaces. Infections caused by biofilm-forming strains of *K. pneumoniae* are difficult to treat (3). 

The increasing rate of multi-drug resistance (MDR) *K. pneumoniae* is a major challenge in hospitals and has been reported in several studies in Iran (4-7). Treatment of MDR* K. pneumoniae* infections is often challenging due to the lack of available therapeutic choices, increasing length of hospitalization morbidity, mortality, and healthcare-associated costs (8*).*

Infections caused by *K. pneumoniae* are mainly treated with beta-lactams and fluoroquinolones (9, 10). Ciprofloxacin belongs to fluoroquinolones used to treat different bacterial infections (10). Resistance to fluoroquinolones is mainly caused by mutations in genes encoding *qyr*A and topoisomerase IV. However, low-level resistance due to plasmid (Plasmid-mediated quinolone resistant=PMQR) is caused by *qnr* genes, QepA, and OqxAB efflux pumps (11-14).

Efflux pumps are found in almost all species of bacteria and play a role in the intrinsic and acquired resistance to many antibiotics. Most of the efflux pump genes are located on the chromosome, but some of them are located on the plasmid. In 2004, efflux pump OqxAB was detected in plasmid-encoded multi-drug resistance in *Escherichia coli* in Denmark. In recent years, there have been reports of increased prevalence of efflux pump OqxAB in the Enterobacteriaceae family (15). A high prevalence of OqxAB efflux pump has been reported in *K. pneumoniae*. The main genetic origin of this efflux pump has been detected in the* K. pneumoniae* chromosome. In several studies, this efflux pump has only been reported in clinical isolates of *K. pneumoniae*, and it has been hypothesized that this efflux pump is widely present among ESBL (extended-spectrum beta-lactamase) producing and carbapenemase-producing isolates (15-18).

A repetitive element palindromic (REP) PCR method such as ERIC-PCR is a quick, reliable, and cost-effective technique for molecular typing of the *Enterobacteriaceae* family, therefore we used ERIC-PCR for the analysis of genetic linkage among *K. pneumoniae* isolates (19).

The main purpose of this study was to investigate the frequency and expression of OqxAB efflux pump genes as well as antibiotic resistance patterns especially ciprofloxacin and molecular typing of *K. pneumoniae* isolates from clinical samples of patients in Hamadan hospitals, west of Iran. 

## Materials and Methods

***Bacterial isolation and identification***

In a cross-sectional study, a total of 100 *K. pneumoniae* isolates were isolated from different clinical samples of patients in different wards of three major hospitals in Hamadan city, from January to June 2021. *K. pneumoniae* isolates were confirmed by conventional and molecular methods (20). 

***Antimicrobial susceptibility testing***

Antimicrobial susceptibility to 12 different antibiotics including amikacin (30 µg), ampicillin-sulbactam (10/10 µg), ceftriaxone (30 µg), cefotaxime (30 µg), ceftazidime (30 µg), ciprofloxacin (5 µg), levofloxacin (5 µg), imipenem (10 µg) meropenem (10 µg), gentamicin (10 µg), nitrofurantoin (300 µg), piperacillin-tazobactam (100/10 µg), tobramycin (10 µg), and chloramphenicol (30 µg) was detected by the disk diffusion method according to CLSI 2021 criteria (21). The antibiotic disks were supplied by Condalab Company from Spain. *Escherichia coli* ATCC 25922 was used as the quality control strain. MICs of ciprofloxacin in *K. pneumoniae* isolates were determined by the micro broth dilution method over a range of dilutions from 0.5 to 256 μg/ml using ciprofloxacin (Sigma–Aldrich). According to CLSI guidelines, breakpoints were used for the interpretation of ciprofloxacin MIC results (susceptible: ≤1 μg/ml; resistant: >1 μg/ml).

***DNA extraction and PCR***

Genomic DNAs of *K. pneumoniae* isolates were extracted by boiling after alkaline (NaOH) treatment of cells (22). Molecular detection of *oqx*A and *oqx*B genes encoding the OqxAB efflux pump was performed by PCR and using specific primers as described previously (23). Amplification reactions were performed in a final volume of 25 μl including 12 μl of 2X master mix (Ampliqon, Demark), 0.5 μM each primer, 2 μl DNA template, and 13 μl of double-distilled water (ddH2O). The PCR conditions were as follows: *oqx*A gene: initial denaturation (2 min at 94 ^°^C), followed by 25 cycles of denaturation (15 sec at 94 ^°^C), annealing (30 sec at 56 ^°^C), and extension (1 min at 72 ^°^C), followed by the final extension at 72 ^°^C for 7 min. *oqx*B gene: initial denaturation (2 min at 94 ^°^C), followed by 32 cycles of denaturation (30 sec at 94 ^°^C), annealing (30 sec at 55 ^°^C), and extension (1 min at 72 ^°^C), followed by the final extension at 72 ^°^C for 10 min. PCR products were subjected to electrophoresis in 1.2% agarose gel (Invitrogen, US) containing DNA Safe Stain (CinnaGen Co, Iran) at 70 V for 1 hr, and the band patterns were visualized in a Gel Doc.

***RNA Extraction and cDNA synthesis for RT-PCR***

Real-time PCR (RT-PCR) was used to investigate the expression level of the* oqx*A gene. A total of 16 *K. pneumoniae* strains were selected based on ciprofloxacin MICs, MDR phenotype, susceptibility to ciprofloxacin, type of clinical sample, and also the presence of the *oqx*A and *oqx*B genes. Total RNA was extracted using an RNA extraction kit (SinaClone, Iran), and converted into cDNA using the cDNA synthesis kit (AddBio, Korea) according to the manufacturer’s instruction. The quality and purity of the RNA obtained was evaluated using a spectrophotometer. 

***Real-Time PCR reaction***

Real-time quantification of cDNA was carried out in a detection system (Roche, Germany) using the SYBR green PCR master mix. The optimized reaction consisted of a master mix (10X), 1 µl of each primer (10 pmol), 2 µl of cDNA (100 μg/ml), and 6 µl of DEPC water in a total volume of 20 µl. *ure*D gene primer was used as the internal control. Expression values (R) were determined using the ΔΔCt method. Expressions of all genes were calculated using the 2^−ΔΔCt^ method (fold). The real-time PCR procedure was programmed as follows: initial denaturation at 95 ^°^C (15 min) followed by 40 cycles of 94 ^°^C (10 sec), 55 ^°^C (60 sec), 72 ^°^C (30 sec), and melt curve at 60 ^°^C (15 sec) and 94 ^°^C for 15 sec.

The analysis of the results was done using the SPSS software, version 22.0 (IBM Co., Armonk, NY, USA). Categorical variables were compared by the Chi-square or Fisher’s exact test and continuous variables were compared by the Mann-Whitney test. The difference in the expression level of *oqx*A was analyzed using a t-test for two independent means.

***ERIC-PCR***

The ERIC-PCR technique was used for the analysis of the genetic link among 100 *K. pneumoniae* isolates. This technique was carried out in a thermal cycler machine (Bio-Rad, Inc. USA) using the primer ERIC (F):5ʹ-ATG TAA GCT CCT GGG GAT TCAC-3ʹ and ERIC (R): 5ʹ-AAG TAA GTG ACT GGG GTG AGC G3ʹ (Metabion Co, Germany) according to the protocol described previously (24). The electrophoresis of PCR products was done on a 2% agarose gel (Sigma-Aldrich) at 70 V for 1 hr, and the patterns of ERIC bands were visualized on gel documentation. The patterns of ERIC bands were analyzed by an online data analysis service (inslico.ehu.es) which compared the ERIC profiles using the Dice method and clustered by the PGMA program.

## Results

Out of 100 *K. pneumoniae *clinical isolates, 33% were isolated from the urine culture, 31% from the trachea, 13% from the blood culture, 12% from the wound, and 1% from Bronchoalveolar lavage (BAL), *Cerebrospinal fluid* (*CSF*), Pleural fluid, and gastric lavage. Out of 100 *K. pneumoniae*, 52% of *K. pneumoniae *strains were isolated from female and 48% from male patients. In this study, the range age of patients was 20-92 years. The prevalence of* K. pneumoniae* was higher in the age range of 71-80 years (24%). Most (74%) *K. pneumoniae-*positive samples were isolated from patients in the ICUs of the hospitals. 

**Figure 1:**
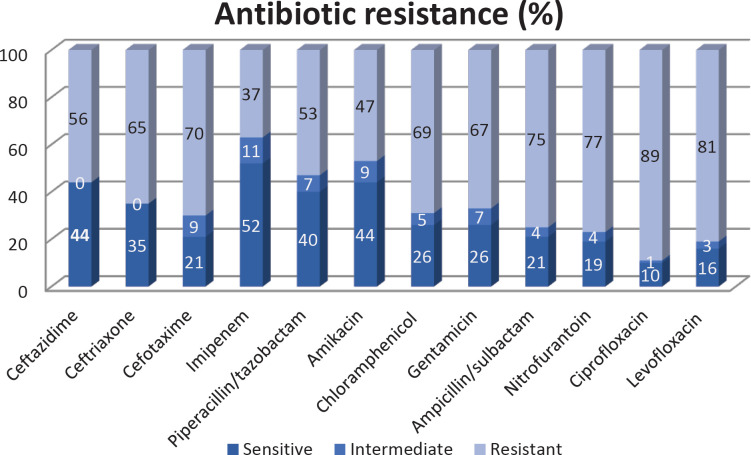
Antibiotic resistance (%) of *Klebsiella pneumoniae* isolated from Hamadan hospitals

According to the results of antimicrobial susceptibility testing by disk diffusion (Figure 1), the highest antibiotic resistance was to ciprofloxacin (89%) and levofloxacin (81%). The MDR phenotype was detected in 65% of isolates. Ciprofloxacin MIC ranged from 0.5 to 256 µg/ml. The MIC ranges of ciprofloxacin were from 0.5-256 µg/ml. MICs equal to 128 µg/ml (18%), 256 µg/lm (26%), 64 µg/lm (23%), 32 µg/ml (18%), 16 µg/ml 16(%), 4 µg/ml (2%), 1 µg/ml (4%), and 0.5 µg/ml (1%) were detected. 

**Figure 2:**
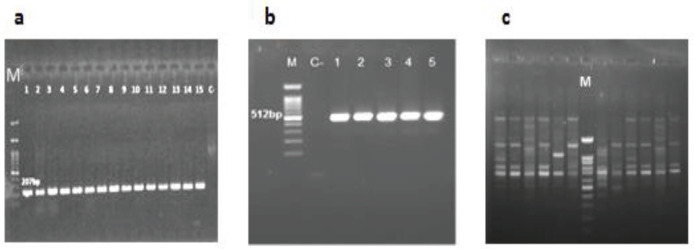
Gel electrophoresis of PCR products of OqxAB encoding genes and ERIC-PCR typing in *Klebsiella pneumoniae* isolates from hospitalized patients in Hamadan hospitals

According to PCR results the prevalence of *oqx*A and *oqx*B was 95% and 98%, respectively (Figure 2). Among the 10 isolates susceptible to ciprofloxacin, 3 (30%) and 1 (10%) of isolates were positive for *oqx*A and *oqx*B, respectively. There was a significant relationship between resistance to ciprofloxacin and *oqx*B gene (*P*-value=0.04) and also between resistance to ceftriaxone (*P*-value=0.01), chloramphenicol (*P*-value= 0.02), and *oqx*A gene. 

**Figure 3:**
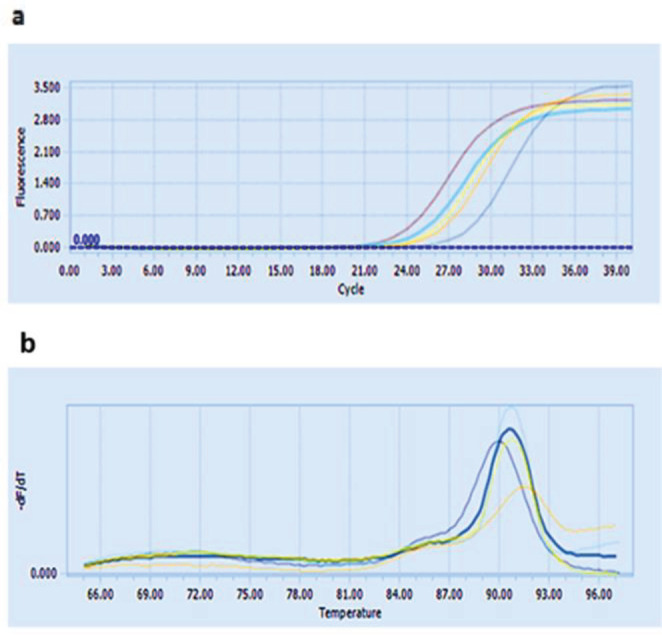
a: Specific amplification of the *oqxA* gene in *Klebsiella pneumoniae *isolates during Real-time PCR, b: melting curve of primers

The results of Real-time PCR showed that the *oqx*A gene was expressed in both ciprofloxacin-susceptible and ciprofloxacin-resistant strains (Figure 3). However, *oqx*A expression was higher in ciprofloxacin-resistant strains than in ciprofloxacin-sensitive strains (Table 1). Statical analysis showed that there was a significant relationship between resistance to ciprofloxacin and *oqx*A gene expression (*P*-value=0.02).

**Figure 4:**
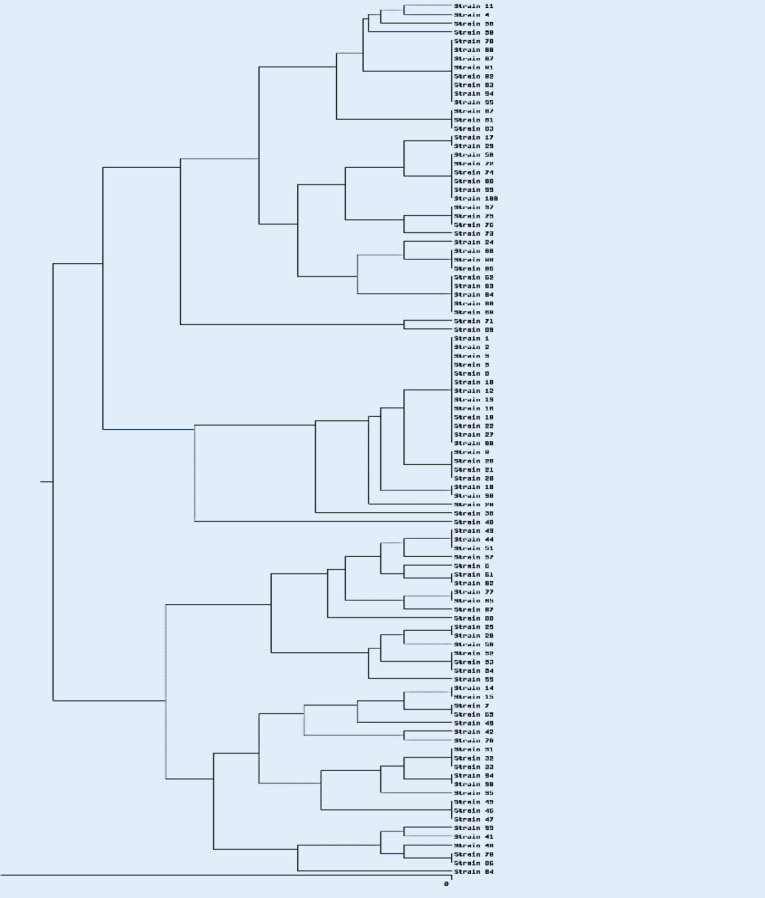
Dendrogram of ERIC-PCR patterns of 100 *Klebsiella pneumoniae* isolates from Hamadan hospitals

Analysis of genetic linkage among isolates by ERIC-PCR showed ≤50-100% similarity among *K. pneumoniae* isolates (Figure 4). Genetic diversity was established among *K. pneumoniae* isolates by detecting 46 different ERIC types with a similarity cutoff ≥95%. 46 different ERIC profiles, including 21 common types (including more than one strain) and 25 unique types (including one strain), were identified. The largest common type includes 13 different strains. These strains were isolated from three different hospitals and different wards which indicates the circulation of clones that are genetically related. There was no significant difference between ERIC groups and antibiotic resistance, hospital, and wards (*P*-value≥0.005). However, most strains in ERIC types were positive for *oqx*A and *oqx*B. The negative *oqx*A and *oqx*B strains were placed in single types and they were placed in no common ERIC type.

a: *oqxA* (207bp), b: *oqxB* (512bp), c: ERIC-PCR, Lane M: 100 bp DNA size marker

**Table 1:** Comparison of gene expression in the studied strains of *Klebsiella pneumoniae*

ΔCT	MIC g/mlµ	MDR	CIP resistance	Sample	Number
4.08	32	+	+	blood	37
3.54	64	+	+	Urine	107
7.2	256	-	+	blood	7
4.58	32	+	+	tracheal	60
4.84	1	+	-	urine	137
2.96	256	-	+	urine	28
5.23	1	+	-	urine	103
7.11	1	-	-	urine	N4
8.62	128	+	+	urine	20
5.57	1	-	-	urine	5
0.26	256	+	+	urine	N5
5.66	64	+	+	sputum	65
1.9	128	+	+	tracheal	N8
4.4	32	+	+	blood	128
7.67	1	+	-	urine	134
0.76	256	+	+	tracheal	4

## Discussion

The results of this study have shown the high prevalence resistance to fluoroquinolones as well as other classes of antibiotics in *K. pneumoniae* isolated from patients in Hamadan hospitals. More than 60% of isolates showed MDR phenotypes. The rate of resistance to ciprofloxacin and levofloxacin in our study is higher than the results of other studies conducted in different regions of Iran (4, 7, 25). A review article reported that the average resistance to ciprofloxacin in Iran was 34.8% from 2002 to 2014 (4). While, according to the results of recent studies, resistance to ciprofloxacin has increased in recent years (6). Resistance to ciprofloxacin is higher than the resistance reported from other countries (26-28). The reason for this difference in the results can be due to differences in the sampling area, population under study, sample size, and the type of strains circulating in different hospitals. The high antibiotic resistance in this study could be due to the fact that most *K. pneumoniae* strains have been isolated from ICUs. The prevalence of resistance to ciprofloxacin and levofloxacin was not significantly different from the earlier study in Hamadan hospitals. In the previous study in Hamadan hospitals, the prevalence of resistance to ciprofloxacin and levofloxacin in *K. pneumoniae* isolates was 80% and 85%, respectively (6). These results demonstrated that appropriate strategies have not been adopted to control or reduce resistant strains in hospitals. The results of our study are significant because our sampling was done during the COVID-19 pandemic; therefore, the presence of highly resistant *K. pneumoniae* strains, especially in ICUs provided a potential threat to patients. 

These results also indicate the lack of appropriate efficacy of these antibiotics against *K. pneumoniae*, which is a worrying result, and physicians should pay more attention to the prescription of these antibiotics for the treatment of patients, especially hospitalized patients, and prescribe antibiotics based on the results of microbiology laboratories. 

In our study, approximately 33% of isolates were related to urine samples, and about 31% of isolates were obtained from tracheal samples. Based on these results, the frequency of urine and respiratory samples is nearly equal or contrary to the results reported by Hamadan hospitals and other studies (24, 29). In the previous study from Hamadan hospitals, about 60% of isolates were related to the respiratory system, and about 20% of isolates were obtained from urine samples (24). In a study from Iran, 70% of *K. pneumoniae* isolates were related to urine samples and 14% were extracted from tracheal samples (29). Nirwati *et al*. isolated 51.5% of *K. pneumoniae* stains from respiratory specimens (30). According to the results of various studies, urinary tract and respiratory tract infections are two major infections in patients that are caused by *K. pneumoniae*.

The detection of *oqx*A and *oqx*B genes in most *K. pneumoniae* isolates was another major finding in this study. Most studies relating to oqxAB have concentrated on the contribution of the OqxAB efflux pump to quinolone resistance. The OqxAB efflux pump is known as one of the mechanisms of resistance to quinolones and fluoroquinolones (31). The OqxAB gene has been commonly detected in quinolone-resistant bacteria such as *E. coli* and *K. pneumonia***e **(31). As demonstrated by Martinez *et al*. the expression levels of OqxAB in *K. pneumoniae* with reduced susceptibility to quinolones were 4-fold higher than in the susceptible strains (11). In our study, resistance to ciprofloxacin was significantly associated with expression levels of the *oqxA* gene. These results indicate a significant relationship between the presence of OqxAB efflux pump genes and resistance to ciprofloxacin. The prevalence of *oqx*A and *oqx*B genes in our study was higher compared with other Iranian studies. (32-34). High resistance to ciprofloxacin may be associated with a high level of efflux pump genes in this study. 

In a report from China, it was found that the OqxAB gene is located in clinical isolates of *Enterobacteriaceae* and *K. pneumoniae* on a chromosome or plasmid. It plays a role in low and moderate resistance to quinoxalines, fluoroquinolones, tigecycline, nitrofurantoin, and many detergents (31). We also identified OqxAB genes in ciprofloxacin-susceptible strains. In this study, there was a significant relationship between resistance to ciprofloxacin and the *oqx*B gene and also between resistance to ceftriaxone and chloramphenicol with the *oqx*A gene. Based on the results of a study nitrofurantoin resistance in *K. pneumoniae* was relatively high; by studying the resistance mechanisms, it was found that the OqxAB efflux pump can play a role in nitrofurantoin resistance (35). Resistance to nitrofurantoin was also high in our study, with 77% of isolates exhibiting resistance to nitrofurantoin. In a study from China, 12 (2%) of 546 human clinical *Salmonella Typhimurium* were co-resistant to both ciprofloxacin and ceftriaxone, and four of the 12 resistant isolates carried the OqxAB gene (36). Based on the results of our study and other studies, the OqxAB efflux pump is not only associated with resistance to fluoroquinolones but can also be associated with resistance to other antibiotics. The high prevalence of *K. pneumoniae* strains producing efflux pump genes containing OqxAB genes has been considered an important issue in hospitals which causes serious problems for infection control and antibiotic treatment. High expression of OqxAB pump seems to contribute OqxAB to ciprofloxacin resistance in *K. pneumoniae*. The presence of OqxAB efflux genes and resistance to antibiotics were also significantly correlated. Considering the possibility of efflux pump genes on plasmids and transferring them to other bacteria, controlling resistant strains and inhibiting the efflux pumps of bacteria is very important. According to these results, the use of natural efflux pump inhibitors in clinical cases and chemical inhibitor efflux pumps in environmental cases in bacteria as an alternative or antibiotic supplement can be beneficial and effective in reducing the incidence of antibiotic resistance, reducing hospitalization time, and also reducing mortality.

The results indicated that 46 different types of ERIC were detected by ERIC-PCR, demonstrating the genetic diversity of *K. pneumoniae* isolates in Hamadan hospitals. Our findings indicated that there was no significant relationship between the ERIC groups and antibiotic resistance patterns, and the OqxAB efflux pump genes. Studies in Iran and other countries have demonstrated the genetic diversity of *K. pneumoniae* isolates according to the PCR-ERIC analysis (20, 23, 28). Our results indicate various *K. pneumoniae* colons with different antibiotic resistance profiles that may cause problems controlling and treating *K. pneumoniae *infections in hospitals.

## Conclusion

To conclude, the prevalence of OqxAB efflux pump is high in *K. pneumoniae* in Hamadan hospitals and constitutes a potential reservoir for the spread of OqxAB among nosocomial bacteria. The high expression of this pump appears to reduce the sensitivity of clinical *K. pneumoniae* isolates to fluoroquinolones. Further studies on the various mechanisms of antibiotic resistance as well as efflux systems are suggested. 

## Authors’ Contributions

LSH and MRA Conceived and designed the experiments. FA Performed the experiments and analyzed the data. LSH and FA Prepared figures and tables and wrote the manuscript. MRA and LSH critically reviewed the manuscript. All authors have read and approved the final manuscript.

## Ethical Approval

The present study was ethically approved by the Institutional Review Board of Hamadan University of Medical Sciences (IR.UMSHA.REC.1399.980).

## Conflicts of Interest

The authors declare that they have no conflicts of interest.
